# Biomarker discovery across annotated and unannotated microarray datasets using semi-supervised learning

**DOI:** 10.1186/1471-2164-9-S2-S7

**Published:** 2008-09-16

**Authors:** Cole Harris, Noushin Ghaffari

**Affiliations:** 1 Exagen Diagnostics, Inc. Houston, TX, USA; 2 Department of Electrical and Computer Engineering, Texas A&M University, College Station, TX, USA

## Abstract

The growing body of DNA microarray data has the potential to advance our understanding of the molecular basis of disease. However annotating microarray datasets with clinically useful information is not always possible, as this often requires access to detailed patient records. In this study we introduce GLAD, a new Semi-Supervised Learning (SSL) method for combining independent annotated datasets and unannotated datasets with the aim of identifying more robust sample classifiers.

In our method, independent models are developed using subsets of genes for the annotated and unannotated datasets. These models are evaluated according to a scoring function that incorporates terms for classification accuracy on annotated data, and relative cluster separation in unannotated data. Improved models are iteratively generated using a genetic algorithm feature selection technique.

Our results show that the addition of unannotated data into training, significantly improves classifier robustness.

## Background

The introduction of DNA microarray technology in 1995 [1] has likely resulted in a huge volume of as yet undiscovered and potentially medically useful knowledge within gene expression profiles. This new bank of information has motivated researchers to develop new techniques for extracting this knowledge, and relating it to externally obtained sample information. For experiments aimed at answering a clinical question, such information might include patient disease stage, or response to a particular drug. The cost of producing adequately annotated datasets has been a barrier to the widespread application of microarray technology in medicine.

Based on the nature of the datasets, a variety of machine learning techniques, including supervised learning algorithms such as classification, and unsupervised learning algorithms such as clustering, have been applied. Clustering techniques [2] are applied to the datasets for assigning samples to their corresponding group solely based on similar expression levels. Supervised algorithms on the other hand classify [3] samples according to their externally determined class.

None of the standard supervised and unsupervised techniques are appropriate for datasets with some unlabeled samples; Semi-supervised algorithms can address these situations.

### Related work

Blum and Mitchell [4] introduced the *co-training *algorithm for improving the sample classification performance when there are few labeled samples and many unlabeled samples. The co-training algorithm assumes that there are two independent sets of features available, such that each feature set is good enough to train a good classifier. The algorithm incorporates an iterative classification of samples from the unlabeled data using two naive Bayes classifiers designed from the independent features sets. In a demonstration of their technique aimed at web page classification, the addition of unlabeled samples decreased classification error relative to classification using only labeled data.

In a subsequent study, Nigam and Ghani [5] further examined the performance of the co-training algorithm and specifically its sensitivity to the independence of the feature sets. Their results confirm that when there is natural split of the features sets, co-training outperforms the other approaches such as expectation-maximization (EM). In the situation that such a split is not available, a random assignment of features into two sets still performs better than using only one feature set. They also introduced the *co-EM *algorithm, a hybrid that iteratively updates the unlabeled data labels using EM. Li et al. [6] proposed a Semi-Supervised Learning (SSL) algorithm for heterogeneous datasets having both labeled and unlabeled samples. Their example data were comprised of DNA microarray expressions and phylogenic reconstructions, with class labels corresponding to gene function. Their work may be considered a form of co-training in that two distinct datasets from a common set of samples (genes) is equivalent to a single dataset with two distinct sets of features. As with the above approaches, independent models are developed for each dataset. They show that minimizing the disagreement in predictions between these models leads to improved accuracy, and introduced a *co-updating *technique for iteratively improving prediction concordance.

Recently, Qi et al. [7] introduced a Bayesian Semi-Supervised approach termed BGEN (Bayesian GENeralization). The BGEN method trains a kernel classifier using both labeled and unlabeled data. Their example data consisted of expression profiles of wild type and mutant *C. elegant *embryos and identified enriched genes, with a small subset of genes labeled according to involvement in development of cell lineage. BGEN predictions were more accurate than predictions from either K-means clustering or SVM classification.

In this paper we propose the Genetic Learning Across Datasets concept (GLAD), and demonstrate an implementation that enables feature selection across unlabeled and labeled datasets. GLAD algorithms are distinct from previous approaches of semi-supervised learning in that the datasets analyzed may have very different statistical distributions, such as would arise in datasets collected independently by labs using different measurement technology. Additionally, a subset of labeled examples is not required for each dataset. As many available datasets will not have the desired annotation for any samples, this method extends the usability of the limited number of adequately annotated microarray datasets.

## Methods

### Datasets

We conducted three experiments, each addressing a different cancer diagnostic problem: ALL/AML differential diagnosis, prediction of response to imatinib in CML, and prediction of outcome in DLBCL. In each experimental group, two microarray gene expression datasets were selected. If available, labels were removed from one of the component datasets, thus creating a combined dataset with both labeled and unlabeled subsets.

All datasets were produced using Affymetrix GeneChips, and in two cases the labeled and unlabeled datasets were collected with different Affymetrix GeneChips. This required mapping of features between the chips in order to identify a common set of features between the chips. GenBank Gene Accession Numbers were used to generate the common features. Table 1 provides additional details on these datasets.

**Table 1:** Dataset details

	**Dataset**	**Genes before mapping**	**Genes after mapping**	**Samples**
**AML – ALL**				

Labeled	AML-ALL 1 [3]	7129	6002	Train: 1-ALL (27) 2-AML (11)
				Test: 1-ALL (20) 2-AML (14)
Unlabeled	AML-ALL 2 [8]	12582	6002	1-ALL (24)
				2-AML (28)
				3-MLL (20: deleted)

**CML**				

Labeled	CML 1 [9]	22283	22283	1-no cytogenetic response to imatinib (15)
				2-cytogenetic response to imatinib (30)
Unlabeled	CML 2 [10]	22283	22283	1-Aggressive (10)
				2-indolent (9)

**DLBCL**				

Labeled	DLBCL 1 [11]	7129	1117	1-DLBCL (32: cured, 26: fatal or refractory)
				2-FL (19: deleted)
Unlabeled	DLBCL 2 [12]	44928	1117	1-DLBCL (176)
				2-MLBCL (34: deleted)

### Demonstrations

For this study we implemented a GLAD algorithm as a wrapper technique for feature selection. A Genetic Algorithm (GA) is used for generating a population of relevant feature subsets. For a given subset, a model is computed from the labeled data and separately for the unlabeled data. Linear Discriminant Analysis (LDA) and K-means (K = 2) cluster algorithms were used for these two data types. A unique two-term scoring function was derived to independently score the labeled and unlabeled data models. An overall score is computed as a weighted average of the two terms as shown below.

*Score *= *w *× *Score*_labeled_+ (1-*w*) × *Score*_*unlabeled*_

We defined the labeled data model score as the standard leave-one-out-cross-validation accuracy for the labeled training samples.

The unlabeled data model score consists of two terms: A cluster separation term and a consistent proportion term.

$$Score_{unlabeled}=\frac{\sum_{i\neq j}\left|C_{i}-C_{j}\right|}{\sum_{i\neq j}\left|C_{i}-C_{j}\right|+\sum_{i}\frac{1}{N_{C_{i}}}\sum_{j}\left|{\text{x}}_{\text{ij}}-C_{i}\right|}-\sqrt{\frac{1}{n_{c}}\sum_{i}{\left(\pi_{i}-\pi_{\exp_{i}}\right)}^{2}}$$

*C*_i _≡ centroid of cluster i

*π*_*i *_≡ proportion of data in cluster i

$\pi_{\exp_{\text{i}}}$ ≡ expected proportion in cluster i

${\text{N}}_{{\text{C}}_{\text{i}}}$ ≡ number of datapoints in cluster i

n_c _≡ number of clusters

The cluster separation term is given by a modified ratio of the inter-cluster distance to the mean cluster size. The consistent proportion term, is defined as the RMS difference between the sorted actual and expected class priors. The class priors may be estimated from the labeled data, or may be available externally.

For each experiment, we did the following:

1. Iterate GLAD algorithm on labeled training data only.

2. Iterate GLAD algorithm on labeled and unlabeled training data.

3. Compare model accuracy on test data across generated populations of models.

In these experiments, GLAD was run for 100 iterations with a population size of 5000, and a subset size of 3 features.

## Results and discussion

**Figure 1:**
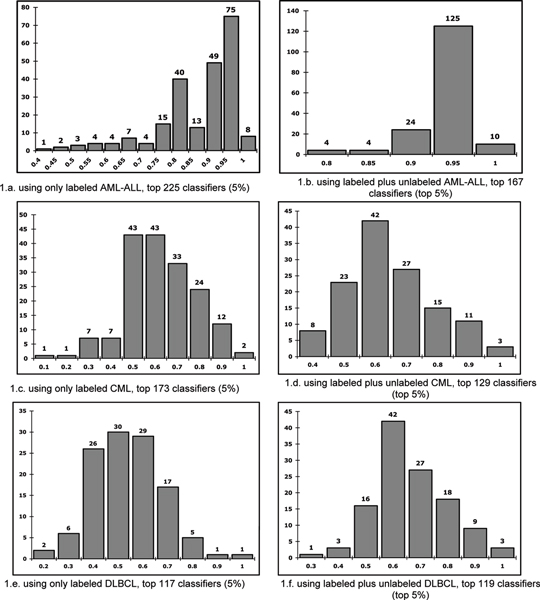
This figures shows the improvement of the classification by adding unlabeled samples into the experiments.

In the first evaluation of GLAD performance, test data classification accuracy was compared between models identified using only labeled data and models using both labeled and unlabeled data. Figure 1 shows the results for three cancer groups. The top 5% of the model populations were used to generate these histograms. For each cancer set, the two histograms can be compared graphically. As is evident in figure 1, adding unlabeled samples increased the mean accuracy of the models significantly.

**Figure 2:**
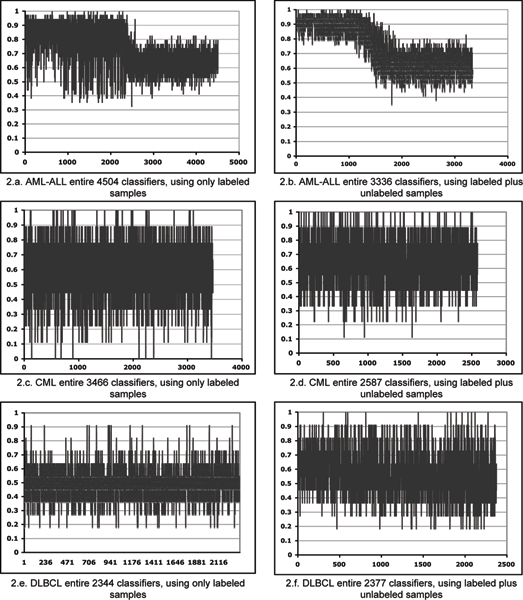
Comparing the performance of the entire unique classifiers on the testing set for two approaches: 1 – using only labeled samples 2 – using labeled plus unlabeled samples.

Figure 2 displays the improvements of the classification accuracies for the population of unique classifiers in each cancer group. The output of GLAD has 5000 models, each comprised of 3 genes, with some duplication of classifiers expected. For testing the classification accuracy on the independent set, only unique classifiers were used. Figure 2 compares the performance of the unique classifiers on the testing set for two approaches: 1 – using only labeled samples 2 – using labeled plus unlabeled samples. The results of the all three cancer groups are improved by adding unlabeled samples to the training sets. Tables 2, 3, 4 show the improvements in more detail. In the AML-ALL group, for the top 1000 classifiers, the accuracy range using only labeled samples is ~40% to 100%. The addition of unlabeled samples increases the range from 70% to 100%. In CML experiments, adding unlabeled samples increases the minimum accuracy from 0% to 11.11%. Combining labeled and unlabeled sample for DLBCL increases the maximum accuracy from 90% to 100%.

**Table 2:** Improvements by adding unlabeled samples for AML-ALL

**Dataset: AML-ALL**	**Unique Classifiers**	**Min**	**Max**	**Average**
only labeled	4504	32.35%	100.00%	73.46%
labeled + unlabeled	3336	35.29%	100.00%	75.14%

**Table 3:** Improvements by adding unlabeled samples for CML

**Dataset: CML**	**Unique Classifiers**	**Min**	**Max**	**Average**
only labeled	3466	0.00%	100.00%	59.34%
labeled + unlabeled	2587	11.11%	100.00%	65.57%

**Table 4:** Improvements by adding unlabeled samples for DLBCL

**Dataset: DLBCL**	**Unique classifiers**	**Min**	**Max**	**Average**
only labeled	2344	18.18%	90.91%	49.67%
labeled + unlabeled	2377	18.18%	100.00%	55.79%

## Conclusion

In this study we proposed a new technique for concurrently mining labeled and unlabeled datasets. This method supplements standard supervised learning with clustering of data lacking clinical annotation to estimate the predictive power of gene subsets. The performance of our algorithm was evaluated in comparison with supervised learning only on microarray data from three different cancer types. Our results show that adding unlabeled samples can increase the accuracy of classification significantly.

## Competing interests

CH and NG were employees of Exagen Diagnostics during the course of this research and the preparation of this manuscript. Additionally, CH owns stock in Exagen Diagnostics.

## Authors' contributions

CH devised and implemented the GLAD algorithm, and contributed to the final preparation of the manuscript. NG ran the experiments, interpreted the results, composed early draft versions of the manuscript and contributed to the final preparation of the manuscript. Both authors read and approved the final manuscript.
